# Effects of Mg Doping at Different Positions in Li-Rich Mn-Based Cathode Material on Electrochemical Performance

**DOI:** 10.3390/nano12010156

**Published:** 2022-01-03

**Authors:** Elena Makhonina, Lidia Pechen, Anna Medvedeva, Yury Politov, Aleksander Rumyantsev, Yury Koshtyal, Vyacheslav Volkov, Alexander Goloveshkin, Igor Eremenko

**Affiliations:** 1 Kurnakov Institute of General and Inorganic Chemistry, Russian Academy of Sciences, 31 Leninsky pr., 119991 Moscow, Russia; lidia.s.maslennikova@gmail.com (L.P.); anna.ev.medvedeva@gmail.com (A.M.); polserv@mail.ru (Y.P.); svolkov77@gmail.com (V.V.); 2 Ioffe Institute, Russian Academy of Sciences, 26 Politekhnicheskaya ul., 194021 St. Petersburg, Russia; rumyantsev.amr@gmail.com (A.R.); yury.koshtyal@gmail.com (Y.K.); 3 Nesmeyanov Institute of Organoelement Compounds, Russian Academy of Sciences, 28 Vavilova ul., 119334 Moscow, Russia; golov-1@mail.ru

**Keywords:** Li-ion battery, cathode material, Li-rich oxide, Mg doping

## Abstract

Li-rich Mn-based layered oxides are among the most promising cathode materials for next-generation lithium-ion batteries, yet they suffer from capacity fading and voltage decay during cycling. The electrochemical performance of the material can be improved by doping with Mg. However, the effect of Mg doping at different positions (lithium or transition metals) remains unclear. Li_1.2_Mn_0.54_Ni_0.13_Co_0.13_O_2_ (LR) was synthesized by coprecipitation followed by a solid-state reaction. The coprecipitation stage was used to introduce Mg in TM layers (sample LR-Mg), and the solid-state reaction (st) was used to dope Mg in Li layers (LR-Mg(st)). The presence of magnesium at different positions was confirmed by XRD, XPS, and electrochemical studies. The investigations have shown that the introduction of Mg in TM layers is preferable in terms of the electrochemical performance. The sample doped with Mg at the TM positions shows better cyclability and higher discharge capacity than the undoped sample. The poor electrochemical properties of the sample doped with Mg at Li positions are due to the kinetic hindrance of oxidation of the manganese-containing species formed after activation of the Li_2_MnO_3_ component of the composite oxide. The oxide LR-Mg(st) demonstrates the lowest lithium-ion diffusion coefficient and the greatest polarization resistance compared to LR and LR-Mg.

## 1. Introduction

Lithium-ion batteries (LIBs) now dominate in portable electronic devices such as mobile phones, computers, cameras, etc., and the portable electronics market constantly grows. By 2030, the stationary and transportation energy storage markets combined are estimated to grow 2.5–4 terawatt-hours annually, approximately three to five times the current 800-gigawatt-hour market [1]. LIBs are considered to capture the majority of energy storage growth in all markets over at least the next 10 years. To meet those needs, especially for their use in batteries for electrical grids and hybrid and electric vehicles, current LIBs need first of all a higher energy density, cycling stability and safety. Cathode materials play a key role in the high-energy battery electrochemical performance. Among the wide variety of positive electrode materials for LIBs, only a few show enough potential for commercialization, and Li-rich and Ni-rich materials are clearly the most promising of those [2,3].

Lithium-excess transition metal (TM) oxides xLi_2_MnO_3_·(1−x)LiMn_a_Ni_b_Co_c_O_2_ with a layered structure, so called Li-rich oxides, are considered to be attractive cathode materials because of their high specific capacity at the upper voltage >4.5 V (>230–250 mA h/g) [4,5,6,7]. The Li_2_MnO_3_ component of Li-rich oxides has a unique oxygen redox activity that has attracted many researchers [8,9]. The anionic redox activity is due to overlapping atomic TM d orbitals and non-bonding O 2p orbitals, which can deliver extra electrons to the cationic TM^(n+1)+^/TM^n+^ redox center [8,10]. Compared to the axial Li−O−TM configurations centered on each O in LiCoO_2_ or LiMn_a_Ni_b_Co_c_O_2_ (NMC), the octahedral O in Li-rich NMC additionally has an axial Li−O−Li configuration. Extraction of the labile oxygen electrons along the Li–O–Li configuration is the origin of oxygen oxidation and extra capacity beyond the TM redox capacity [11]. However, the oxygen redox reaction in Li-rich layered oxides usually results in O–O dimerization (2O^2−^→O^2n−^) [12,13]. This leads to O_2_ release, the formation of oxygen vacancies, and the migration of transition metal ions during charge–discharge, resulting in a poor cyclability, voltage decay, and safety problems for high-energy LIBs [14,15]. The migration of transition metals to the Li-layer hinders Li+ reinsertion and causes a large voltage hysteresis between charge and discharge [16,17]. To suppress the process of O–O dimerization and gas release from the surface, many efforts have been undertaken, including surface coating [18], surface modification [19,20], element doping and co-doping with different elements [21], simultaneous surface coating and doping [22,23], and local symmetry tuning [24].

Doping seems to be one of the most effective modification methods to change the local atomic environment to consequently affect the oxygen redox reaction and electrochemical performance of Li-rich cathode materials. The doping of Li-rich oxides with Na, K, Mg, Al, Fe, Co, Ru, and other elements have been found to stabilize capacity and mitigate the discharge-voltage decay in these materials [14,25]. There are several studies dealing with the effects of doping Li-rich oxides with Mg. Considering that matching the ionic radii, Mg^2+^ with the ionic radius similar to that of Li^+^ and larger than those of transition metal ions (0.69 Å, 0.545 Å, and 0.53 Å for Ni^2+^, Co^3+^, and Mn^4+^, respectively) would be more suitable to occupy the sites in Li layers rather than in TM layers. Nevertheless, the authors of [26] have been studied the effect of 10 Li-site cationic dopants (Mg, Ti, V, Nb, Fe, Ru, Co, Ni, Cu, Al) on the electrochemical properties of Li_2_MnO_3_ and LiMnO_2_ using density functional theory (DFT). According to the DFT calculations, Mn sites are thermodynamically favorable over Li-site doping. However, the small thermodynamic barriers between both configurations can be easily overcome during the material synthesis and/or the extraction/insertion of Li during the cycling process of the battery. Indeed, the examples of doping magnesium at both the Li and TM positions are known, such as the studies [27,28], where Mg was doped at Li positions and [29,30,31], where Mg was doped at TM positions. Mainly, Mg doping leads to a better cyclability of Li-rich oxides, regardless of the substituted position. At the same time, it has been reported that site-dependent doping has different impacts on the electrochemical properties of cathode materials [32]. It is of interest to compare the Li-rich oxides of the same composition doped with Mg at different positions.

In this work, we studied Mg-doped Li-rich layered cathode materials synthesized by different procedures with the aim to introduce Mg ions at different positions, namely, in TM- and Li layers, and compare their electrochemical properties. The oxides were synthesized by coprecipitation followed by a solid-state reaction with a lithium source. Magnesium (2 mol % of the Co amount) was added at the precipitation step (LR-Mg oxide) and in the solid-state reaction (2 mol % of the Li amount, LR-Mg(st) oxide). For comparison, Li-rich oxide of the same composition except magnesium, Li_1.2_Mn_0.54_Ni_0.13_Co_0.13_O_2_ (LR), was obtained. The materials were characterized by X-ray powder diffraction (XRD) with Rietveld refinement, scanning electron microscopy (SEM) with local X-ray energy dispersive spectroscopy (EDX), transmission electron microscopy (TEM) with selected-area electron diffraction (SAED), X-ray photoelectron spectroscopy (XPS), Brunauer–Emmett–Teller surface area measurements, charge–discharge tests, and galvanostatic intermittent titration (GITT). The LR-Mg and LR-Mg(st) oxides reveal different behavior when tested as half-cell Li-ion cathodes. LR-Mg shows a better cyclability and rate performance than LR-Mg(st) or the reference sample.

## 2. Materials and Methods

### 2.1. Synthesis

The Li-rich oxide materials were synthesized by coprecipitation followed by a solid-state reaction with a lithium source. The starting reagents for all synthetic procedures were Ni(NO_3_)_2_∙6H_2_O (99.9%, ABCR, Karlsruhe, Germany), Co(NO_3_)_2_∙6H_2_O (99%, Acros Organics, Geel, Belgium), Mn(NO_3_)_2_∙4H_2_O (98% Alfa Aesar, Kandel, Germany), and Mg(NO_3_)_2_∙6H_2_O (98%, Alfa Aesar), LiOH∙H_2_O (99+%, Sigma-Aldrich, Saint Louis, MO, USA), MgO (97%, Chimmed, Moscow, Russia), Na_2_CO_3_ (98%, Chimmed), NH_4_OH (98%, Chimmed). All the reagents were used as received. For the precursor synthesis, a mixed aqueous solution of manganese, nickel and cobalt nitrates in appropriate amounts (including magnesium nitrate for the synthesis of LR-Mg sample) with a total concentration of 2.0 mol L^−1^ was prepared. The solution obtained and an aqueous solution of a precipitator (Na_2_CO_3_, 2.0 mol L^−1^ and NH_4_OH, 0.3 mol L^−1^) were added simultaneously into a reactor using peristaltic pumps. The synthesis was carried out under a CO_2_ atmosphere. The pH value, reaction temperature, and stirring speed were kept at 7.5, 60 °C, and 1000 rpm, respectively. The precursor suspension thus obtained was stored for 15–16 h. Then, the carbonate precursor was washed with deionized water, acetone, and diethyl ether, dried at 110°C in an argon flow for 18–20 h, and thoroughly mixed with LiOH·H_2_O in an appropriate amount. A lithium excess (3 mol %) was used to compensate for lithium losses during sintering. For the synthesis of LR-Mg(st) oxide, MgO was added at the solid-state stage. The powders obtained were annealed in air at 480°C for 6 h and at 900°C for 12 h. In the course of annealing, the mixtures were ground several times in an agate mortar.

### 2.2. Methods

The morphology, microstructure and element distribution of the Li-rich materials were studied using scanning electron microscopy (SEM, NVision-40, Carl Zeiss, Oberkochen, Germany) with energy dispersive X-ray spectroscopy (EDX) and transmission electron microscopy (TEM, JEOL JEM 2100, Peabody, MA, USA, E = 200 kV) with EDX analysis. Selected-area electron diffractions (SAED) were taken. Particle size distribution was measured with the use of an Analysette 22 MicroTec Plus laser particle sizer (Idar-Oberstein, Germany). The Fraunhofer model was used for data analysis. The metal contents in the oxides prepared were measured by inductively coupled plasma mass-spectrometry (ICP-MS) with an Agilent 7500ce system (Santa Clara, CA, USA).

The X-ray powder diffraction (XRD) measurements were performed at room temperature in the Θ/2Θ scan mode (2Θ range of 5°–80°, 0.02° scan step) using diffractometers Bruker D8 Advance (CuKα radiation) and Bruker D8 Advance Vario (CuKα1, Ge monochromator, LynxEye position-sensitive detector, Billerica, MA, USA). The data were collected with the use of the Bruker DIFFRACplus program package. The analysis was performed with the EVA and TOPAS software packages. The diffraction patterns were refined by the Rietveld method. The crystallite sizes were estimated by using Williamson–Hall analysis.

The X-ray photoelectron spectroscopy (XPS) measurements were performed using a PHI5000 VersaProbe II instrument (Kanagawa, Japan) applying a monochromatic, micro-focused, scanning Al Kα X-ray source (50 Wt, 1486.6 eV) with 200 μm spot size. The K-Alpha charge compensation system was used during analysis. The XPS data were collected from the area of 800 × 400 µm^2^ at a pass energy of 23.5 eV with a step size of 0.2 eV for C1s, O1s, Mn3s; and at a pass energy of 29.35 eV with a step size of 0.25 eV for Mn2p, Co2p3, Ni2p3.

The specific surface areas (*S*_BET_) of the samples were measured by low-temperature nitrogen adsorption with a Katakon ATX-06 analyzer (Novosibirsk, Russia). The samples were degassed at 200 °C in a dry helium flow for 35 min. The specific surface areas of the samples were calculated from the adsorption isotherms using the Brunauer–Emmett–Teller equation (BET).

### 2.3. Electrochemical Measurements

Electrochemical studies were carried out in CR2032 coin-type cells with a Neware BTS CT-4008W-5V10mA battery testing system using at least three to five cells per sample. The cathodes were fabricated from a mixture of the active material, carbon black (Super C65, Timcal, Bodio, Switzerland), and polyvinylidene difluoride (PVDF) as a binder at the ratio of 92:3:5 wt %. *N*-methylpyrrolidone (NMP) was used as a solvent. The active material loadings were equal, averaging 5–7 mg cm^−2^. The separator was Celgard 2325 (2 layers); the negative electrode was made of metallic lithium foil; and the electrolyte was TC-E918 (Tinci, Guangzhou, China). The electrode preparation is described in more detail elsewhere [33]. Galvanostatic charge-discharge tests were carried out in a voltage range of 2.5–4.7 V at room temperature at different current densities. Before cycling in this range, the high-voltage monoclinic phase was activated at a current density of 20 mA/g as follows: two cycles in the range of 2.5–4.3 V, then two cycles at 2.5–4.5 V, and two cycles at 2.5–4.6 V.

Galvanostatic intermittent titration (GITT) was performed during discharge processes to determine the lithium-ion diffusion coefficient (*D_Li+_*), and the polarization (*R_pol_*_._) and ohmic (*R_ohm._*) resistance values. The samples were discharged at a constant current of 100 mA g^−1^ for 8 min, succeeded by the current interruption (relaxation time) for 30 min, which was sufficient to achieve the equilibrium voltage value. *D_Li+_* was determined using the GITT results according to Equation (1), as introduced by Weppner [34,35,36].

(1) DLi+=4πτ(mVmMS)2(ΔEsΔEt)2, (τ⊲⊲L2DLi+)

where:

*D_Li+_*—lithium-ion diffusion coefficient (cm^2^/s);
*τ*—charge/discharge time of one GITT step (s);
*m—*the active material weight (g);
*V_m_*—the molar volume of the sample (cm^3^/mol);
*M—*the atomic weight (g/mol);
S—the area of the sample-electrolyte interface (cm^2^);
∆*E_s_*—the voltage difference between the initial discharge voltage and the voltage after relaxation;
∆*E_t_*—the voltage change during one GITT step;
*L—*the sample thickness (cm).

From the GITT results, we estimated the resistance values by the following procedure. At first, the voltage was measured at the end of each discharge step (*U1*). Then, the cut-off voltage was measured immediately after the current interruption (*U2*), and at the end of the relaxation time (*U3*). The cell resistances were calculated from the voltage differences between *U2* and *U1* (*R_ohm._*), and *U3* and *U2* (*R_pol_*_._) according to Equation (2) and Equation (3), respectively.

(2) $$R_{ohm.}=\frac{U2-U1}{I}$$

(3) $$R_{pol.}=\frac{U3-U2}{I}$$

where:

*I*—discharge current (A).

## 3. Results and Discussion

In the comparative investigation, we used the Li-rich composition Li_1.2_Mn_0.54_Ni_0.13_Co_0.13_O_2_, which can be written in another notation as 0.5Li_2_MnO_3_·0.5LiMO_2_ (M = Mn_0.333_Ni_0.333_Co_0.333_). To obtain LR-Mg oxide, magnesium nitrate was added during the precipitation of the carbonate precursor at the expense of cobalt (2 mol %). To obtain LR-Mg(st), magnesia was added together with lithium hydroxide in the solid-state reaction (2 mol % of the Li amount). The oxide compositions determined by inductively coupled plasma mass spectrometry (ICP-MS) are close to those preset in the synthesis (Table S1).

**Figure 1:**
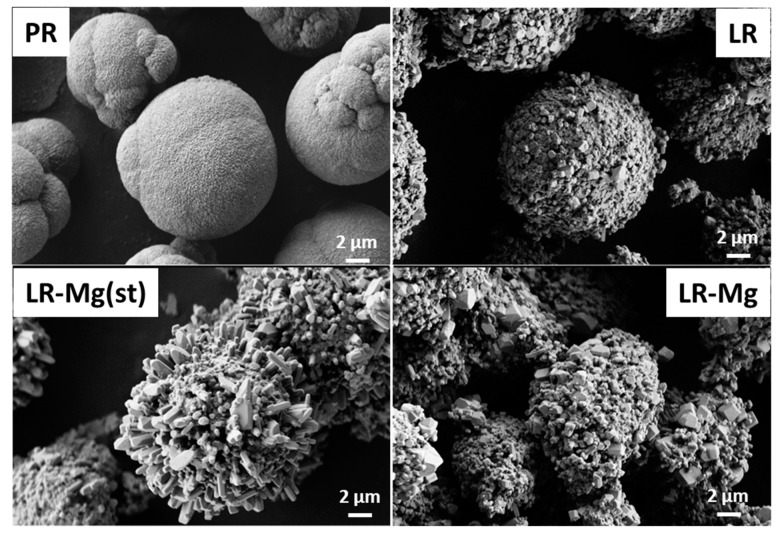
SEM images of the carbonate precursor for LR (PR) and of LR; LR-Mg; and LR-Mg(st) oxides (sample names are shown in the figures).

The SEM images of LR-Mg, LR-Mg(st), LR, and the precursor for LR (PR) are shown in Figure 1.

All the oxides are sphere-like agglomerates consisting of primary particles 200–500 nm in size. The oxides maintained the precursor morphology. LR and LR-Mg oxides have narrow particle size distributions that are very close to each other. The LR-Mg(st) agglomerates and primary particles were characterized by a wider range of sizes (Figure S1). In addition, LR-Mg(st) contains large particles up to 4–5 μm in size. The element distribution EDX maps of LR-Mg and LR-Mg(st) (Figure S2) show that all the elements, including magnesium, were evenly distributed over the particle surface of both oxides.

**Figure 2:**
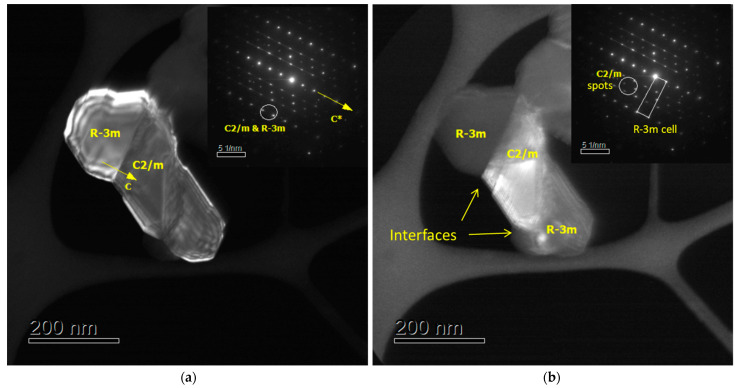
Results of TEM study of the reference sample Li_1.2_Mn_0.54_Ni_0.13_Co_0.13_O_2_ (LR): (**a**,**b**) DF images of a grain with intergrowth of crystals taken with the reflections marked with circles in SAED patterns; SAED patterns along the (100)_m_ zone are shown in the inserts.

The X-ray powder diffraction spectra of the materials obtained were typical for Li-rich oxides [37]. No additional XRD peaks were detected upon Mg doping, indicating the absence of any impurity phase. The XRD peaks can be indexed based on a cell with trigonal ($R\bar{3}m$) or monoclinic (*C*2/*m*) symmetry. According to our previous investigations of Li-rich oxides of the same composition using TEM with local electron diffraction [38], the oxides contained both structurally integrated phases: trigonal and monoclinic. This agreed with a large body of the literature [3,37,39]. Figure 2 presents the data of the TEM/SAED analysis of oxide LR.

Figure 2 demonstrates an evidence of intergrowth of trigonal ($R\bar{3}m$) and monoclinic (*C*2/*m*) phases at nanoscale. Selected-area electron diffractions clearly showed the reflections of monoclinic and trigonal phases. A grain consisted of nanodomains was taken with the reflections marked with circles in the SAED patterns, which are very important to distinguish the $R\bar{3}m$ and *C*2/*m* phases. The weak *C*2/*m* superstructure spots, as observed and encircled in the SAED pattern of Figure 2b, usually appear to be very weak in XRD powder patterns. Therefore, taking into account that the *C*2/*m* group appears to be a subgroup of the $R\bar{3}m$ group, we may try to neglect these superstructure spots by fitting a monoclinic phase with the trigonal $R\bar{3}m$ space group in the simplified XRD Rietveld refinement presented below by using a rhombohedral layered model in the space group $R\bar{3}m$ [40].

**Figure 3:**
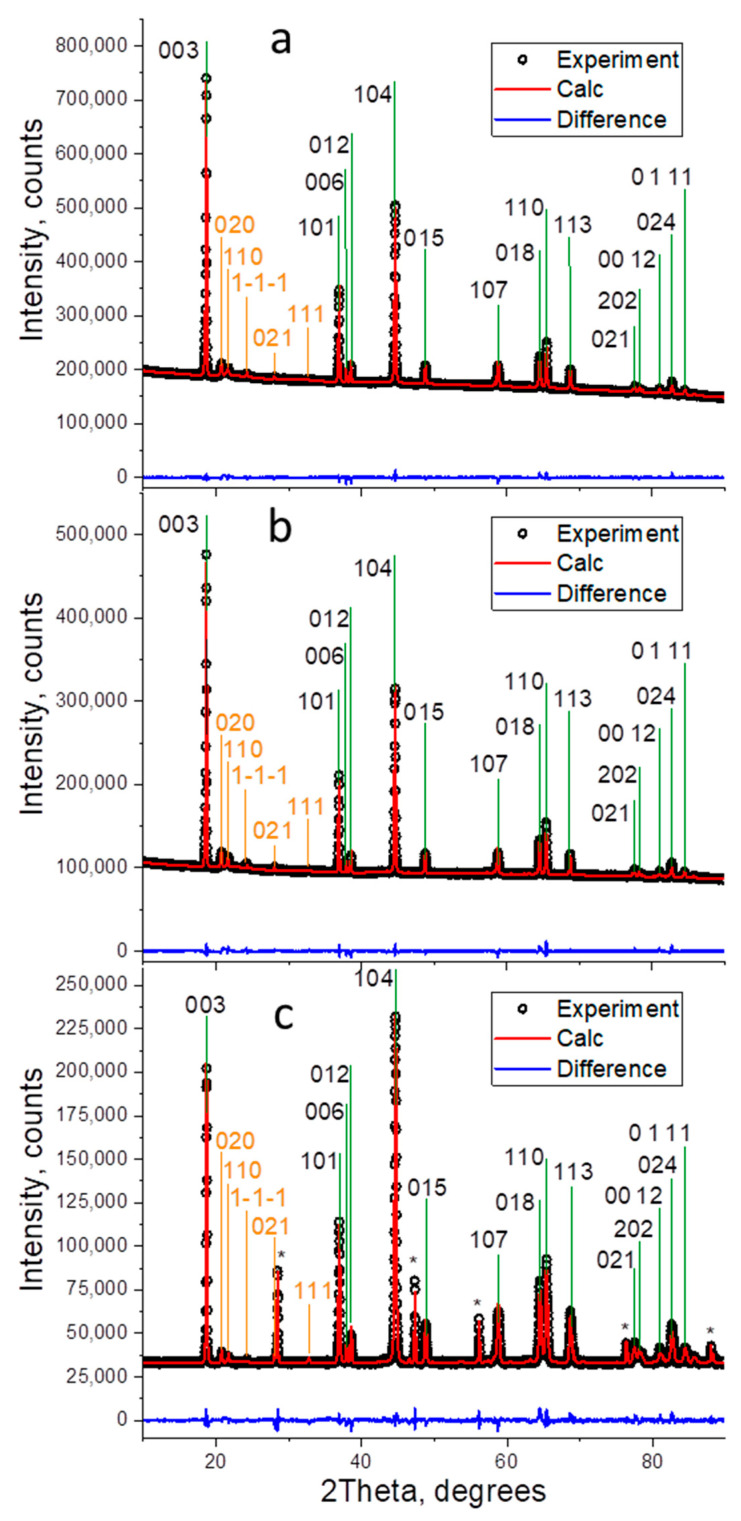
Refined powder X-ray diffraction patterns for materials prepared: (**a**) LR; (**b**) LR-Mg; and (**c**) LR-Mg(st). Diffraction measurements of LR and LR-Mg were carried out with a Ge monochromator; the diffraction pattern of LR-Mg(st) oxide was measured without monochromator using a Si standard reference; the Si lines are designated by asterisks.

Refined powder X-ray diffraction patterns of the oxides under study are shown in Figure 3.

A few broad peaks appeared in the 2Θ range of 20°–25° are associated with the presence of a monoclinic phase. Most of the peaks of the trigonal and monoclinic phases overlap. The peaks of the monoclinic phase, which do not coincide with the trigonal phase peaks have substantial anisotropic broadening so that they should be excluded from the Rietveld refinement because cannot be correctly simulated within this method. Comparison of the intensity ratios of I(003)/I(104) and I(101)/I(104) reflections for the oxides shows that the ratios are nearly the same for LR and LR-Mg (Figure 3a,b), but considerably decreased for LR-Mg(st) (Figure 3c). According to the model calculations, both of these ratios should essentially go down compared to undoped sample LR when any metal will begin occupying the Li-ion layer. On the other hand, these ratios remain very high when the doping metal goes to the transition metal layer, as demonstrated for LR-Mg (Figure 3b). The phase compositions, refined lattice parameters, *R*-factors, and goodness of fit are presented in Table 1.

The ratios between the amounts of the trigonal and monoclinic phases were evaluated to be equal within the margin of error for all the oxides. The lattice parameters (*a*, *c*) and the unit cell volume increase for both Mg-doped samples was compared with the reference oxide LR. The ionic radius of Mg^2+^ (0.72 Å) is larger than that of Co^3+^ (0.54 Å). This increase in the unit cell parameters can be attributed to the introduction of magnesium at the TM positions. In case of Mg doping in Li layers, the ionic radii of Mg^2+^ and Li^+^ (0.76 Å) were close to each other. However, when Mg^2+^ is introduced at lithium sites, some of the TM ions would be partially reduced, accordingly. The reduced TM ions have larger ionic radii than those at higher oxidation states; this also should lead to an increase in the unit cell parameters, which was observed for the LR-Mg(st) sample. Moreover, the introduction of magnesium in the TM layers induces an increase in the oxidation number of the neighboring TM ions in layered structures [41], and the corresponding increase in the lattice parameters may not be very large, which is what we actually observed.

The binding energies (BE) and full widths at half maximum (FWHM) of the XP lines of the LR, LR-Mg, and LR-Mg(st) oxides are presented in Table 2.

The binding energies of manganese and cobalt from the 2p3 regions were identical for all the samples. However, we observed a positive shift and broadening of the XP peak from the Ni2p_3/2_ region in the spectrum of LR-Mg compared to those peaks in the spectra of LR and LR-Mg(st). In both the Ni^2+^ and Ni^3+^ cases, it was difficult to assign a single BE to the definite chemical state. Nevertheless, the observed phenomenon can be indicative of the appearance or increase in the Ni^3+^ content in LR-Mg sample [42]. Since we introduced Mg^2+^ at the Co^3+^ positions in this sample, charge compensation can occur at the expense of increasing Ni^3+^ content. On the other hand, the XP spectra of LR-Mg(st) did not show a significant change in the Ni2p_3/2_ peaks compared to the LR oxide. In this case, as noted above, some TM ions would be instead partially reduced. A similar effect of an increase in the relative ratio of Ni^3+^/Ni^2+^ ions on the surface as a result of a partial substitution of Mg for Ni in LiNi_0.6_Co_0.2_Mn_0.2_O_2_ was observed in [43].

The XRD and XPS data demonstrate different behaviors of Mg-doped oxides and support our assumption that magnesium is introduced at different positions when different synthesis procedures are used.

**Figure 4:**
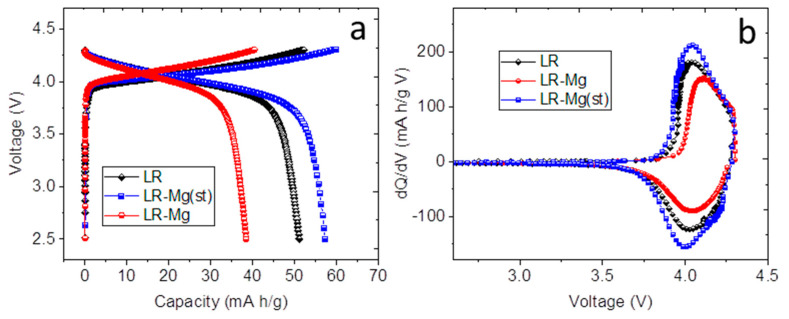
(**a**) The charge-discharge curves and (**b**) the corresponding differential capacity (dQ/dV) plots in the second cycle in the potential range of 2.5–4.3 V. Sample names are shown in the figures.

The oxides were tested in half-cells as cathode materials. Galvanostatic cycling at different current densities and galvanostatic intermittent titration were performed in the range of 2.5–4.7 V. Before cycling, the high-voltage monoclinic phase was activated at a current density of 20 mA/g, as follows: two cycles in the range of 2.5–4.3 V, two cycles at 2.5–4.5 V, and two cycles at 2.5–4.6 V. The charge-discharge curves and the corresponding differential capacity (dQ/dV) plots for the samples in the second charge-discharge cycle (2.5–4.3 V) are shown in Figure 4.

The charge-discharge curves in the range of 2.5–4.3 V (Figure 4a) show different capacities of the samples under study. The electrochemically active species in this voltage range were the trigonal phase LiMO_2_. The positions of the anodic and cathodic peaks in the corresponding dQ/dV plots (Figure 4b) of LR-Mg(st) and LR samples are close to each other, whereas those of LR-Mg are different. This seems to reflect the difference in the compositions of the trigonal phases and indirectly confirms that magnesium enters in the trigonal phase at the TM (Co) positions in LR-Mg oxide. The cause for the lower capacity of LR-Mg oxide charged to 4.3 V may be the smaller content of cobalt in the trigonal phase in this sample. Possibly, there is an additional reason for this capacity decrease. The authors of [44] were unable to extract chemically all the lithium from the LiCo_0.9_Mg_0.1_O_2_ phase and proposed that the presence of Mg^2+^ ions may lead to a trapping or isolation of some Co^3+^ ions as …Mg^2+^–Co^3+^–Mg^2+^…, preventing some of the Co^3+^ ions from participating in the redox process. The same mechanism may be realized in our case. According to the XPS data, LR-Mg contains also a larger concentration of Ni^3+^, which may reduce the specific capacity as well.

**Figure 5:**
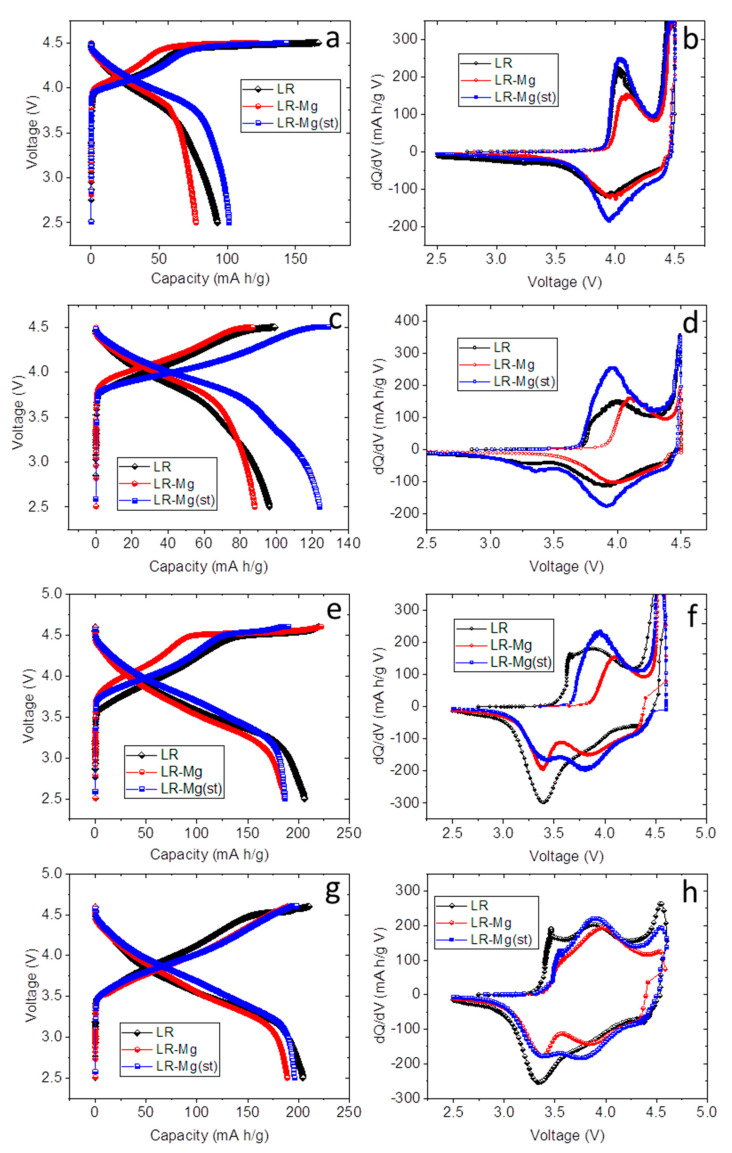
(**a**,**c**,**e**,**g**) The charge-discharge curves and (**b**,**d**,**f**,**h**) the corresponding differential capacity (dQ/dV) plots for the cycles in the potential ranges of (**a**–**d**) 2.5–4.5 V and (**e**–**h**) 2.5–4.6 V. The high peaks at 4.5 V (**b**,**f**) are not shown for clarity. Sample names are shown in the figures.

The first cycles in the wider potential ranges (up to 4.5 and 4.6 V) exhibited the second plateau in the charge-discharge curves and the corresponding peak at ∼4.5 V in the dQ/dV plots (Figure 5), which were attributed to the activation of Li_2_MnO_3_ accompanied by lithium and oxygen loss [6,45].

With increases to upper charge voltage, the difference between the dQ/dV plots of the samples becomes more apparent. All the samples exhibited a new cathodic peak at 3.3–3.5 V and a small anodic peak (or shoulder) at about 3.5–3.7 V in the dQ/dV plots. These peaks were more pronounced for the reference sample LR, whereas both the Mg-doped samples showed more pronounced cathodic peaks representing the reduction of nickel (cobalt) at 3.6–3.7 V.

**Figure 6:**
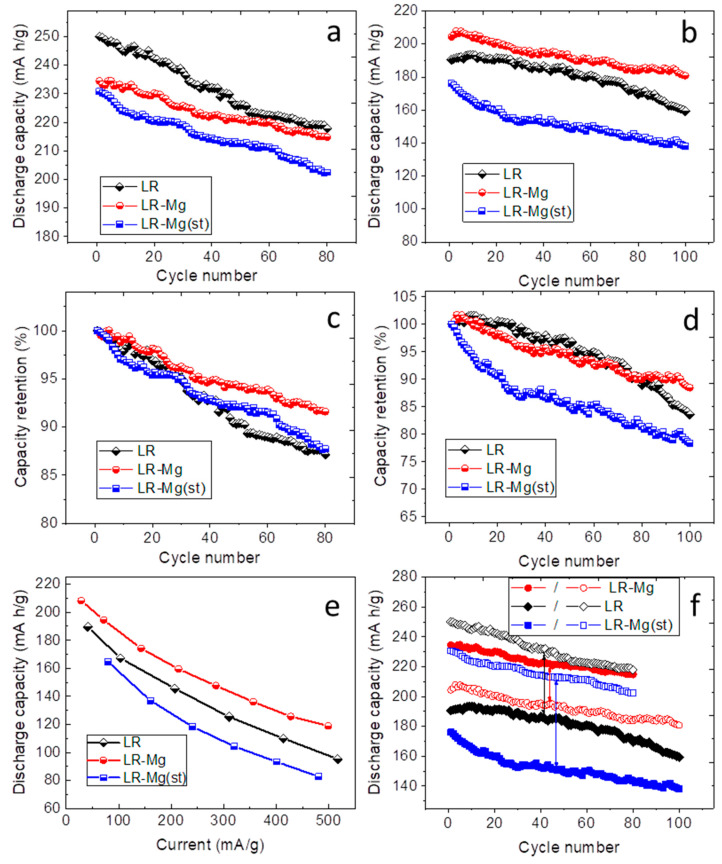
Electrochemical performance of LR, LR-Mg, and LR-Mg(st): discharge capacity as a function of cycle number at the current densities of (**a**) 20 and (**b**) 100 mA/g; capacity retention vs. cycle number at (**c**) 20 and (**d**) 100 mA/g; (**e**) rate performance; and (**f**) comparison of sample cycling performance at different current densities (solid and open symbols correspond to the current densities of 20 and 100 mA/g, respectively).

The electrochemical performance of the samples under study is presented in Figure 6.

**Figure 7:**
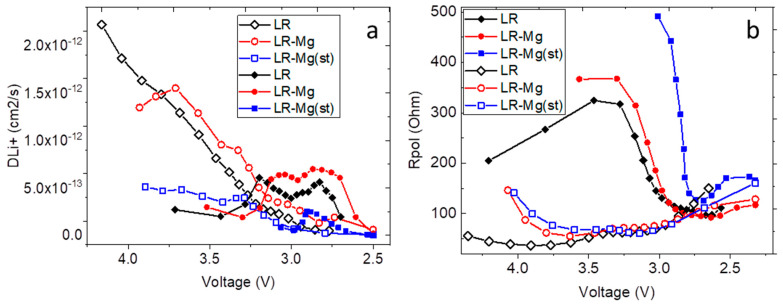
Variation of (**a**) *D_Li+_* and (**b**) *R*_pol_ as a function of cell voltage for different cycles determined by GITT. Open and solid symbols designate the data derived from 11-th and 77-th cycles, respectively. Sample names are shown in the figures.

At the current density of 20 mA/g (corresponds to 0.1C rate), both oxides doped with magnesium show discharge capacities lower than the discharge capacity of the reference sample (Figure 6a). At the current density of 100 mA/g (Figure 6b), the discharge capacity of LR-Mg was higher than that of LR, whereas the discharge capacity of LR-Mg(st) considerably decreased compared with the reference sample. LR-Mg also demonstrates better cyclability at both current densities, which is clearly seen in the capacity retention plots (Figure 6c,d). The cyclability of LR-Mg(st) oxide is nearly the same as that of LR. The rate performance of LR-Mg(st) is also comparable to that of LR, whereas the dependence of the discharge capacity on the current density for LR-Mg is somewhat better than that of LR (Figure 6e). Figure 6f shows a comparison of the discharge plots of the samples at different current densities. One can see that an increase in the current density has the greatest impact on LR-Mg(st) cycling, and the least impact on LR-Mg cycling. One reason for this behavior is the better kinetics in the latter sample. To check this assumption, galvanostatic intermittent titration was performed for all the samples during discharge at a current density of 100 mA/g to determine the lithium-ion diffusion coefficient (*D_Li+_*) and the polarization (*R*_pol_) and ohmic (*R*_ohm_) resistance values [34,35,36]. The GITT measurements were carried out at intervals of 10 cycles. The *D_Li+_* of the oxides as a function of the cell voltage derived for the 11-th and 77-th discharge titration cycles are shown in Figure 7a.

**Figure 8:**
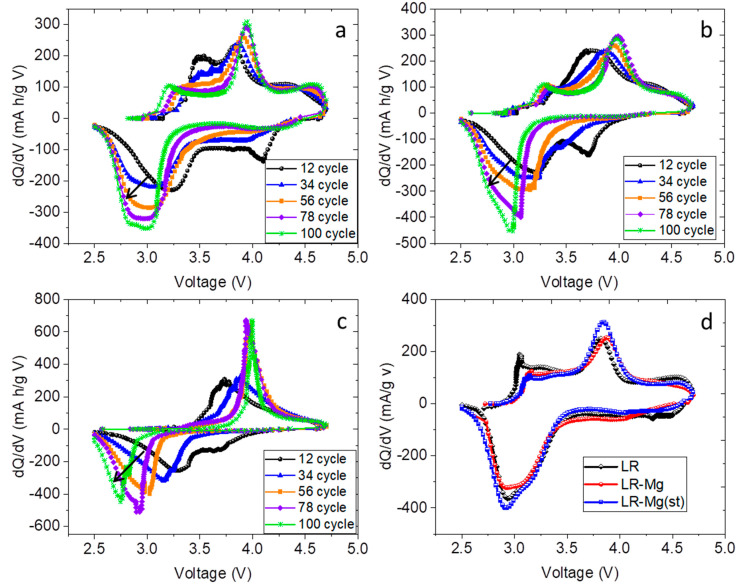
Differential capacity plots (dQ/dV) of the samples (**a**) LR; (**b**) LR-Mg; and (**c**) LR-Mg(st) in different charge-discharge cycles at the current density of 100 mA/g and (**d**) comparison of dQ/dV plots for the samples in the 60th cycle at the current density of 20 mA/g (samples names are shown in this figure).

In both cycles, LR-Mg shows the highest lithium-ion diffusion coefficient, and the *D_Li+_* of LR-Mg(st) was the lowest one. The polarization resistance shows the maximal values also for LR-Mg(st). At the beginning, *D_Li+_* decreases during the discharge process (Figure 7a, open symbols); and *D_Li+_* passes through maxima with increasing cycle number (Figure 7a, solid symbols). The maximum positions roughly corresponded to new peaks appearing in dQ/dV plots in the course of cycling (Figure 8).

The cathodic peaks in the range of 2.8–3.2 V were very broad and can be assigned to different redox species. The overlapping peaks can be interpreted as cathodic peaks due to the reduction of nickel (cobalt) and the peak occurred due to the reduction of manganese-containing redox species [46]. In the literature, the appearance of the reduction peak at a lower potential is commonly associated with an internal phase transition to a spinel-like phase due to the migration of transition metal ions into vacant sites of the lithium layers during the charging process, which leads to voltage decay in the course of cycling [47,48,49,50]. The cathodic peaks shifted to low voltage in the dQ/dV plots with cycling. This shift was the lowest for LR-Mg and the greatest for LR-Mg(st). At the same time, the new phase seems to contribute to the overall discharge capacity. Simultaneously with the appearance of the new cathodic peak, we observed a small anodic peak at about 3.0–3.3 V for LR and LR-Mg oxides. However, this anodic peak was absent in the dQ/dV plots of LR-Mg(st) at the current density of 100 mA/g. A comparison of the dQ/dV plots of all the samples in the 60th cycle at the current density of 20 mA/g are presented in Figure 8d. As is seen, the dQ/dV plots are similar, and a shoulder near 3.0–3.3 V appears in the anodic dQ/dV curve of LR-Mg(st) as well. This indicates that kinetic hindrance exists for oxidation of manganese-containing species in LR-Mg(st) oxide, which decreases the reversibility of the redox reaction of the species. The specific discharge capacity of LR-Mg(st) (Figure 6a,b) is, indeed, significantly lower than that of LR and LR-Mg at the higher current density. The authors of [46] studied the Li-rich oxide Li_1.2_Ni_0.15_Co_0.1_Mn_0.55_O_2_ [0.5Li(Ni_0.375_Co_0.25_Mn_0.375_)O_2_·0.5Li_2_MnO_3_] by a combination of XRD, TEM, conventional and time resolved in situ XAS experiments, and GITT techniques. Based on the EXAFS analysis and changes in the Debye–Waller factors, the authors have shown that Mn sites had much poorer reaction kinetics both before and after the initial activation of Li_2_MnO_3_ compared to Ni and Co in this Li-rich oxide. They deduced also that the severe local structural changes in the Mn–O coordination shell observed during the initial delithiation can be mainly attributed to the Li_2_MnO_3_ component of the Li-rich material as a result of the elimination of Li/Mn ordering in Li_2_MnO_3_ and/or the oxygen release accompanied with lithium extraction. Taking the above into account, we can assume from our results that Mg^2+^ substitutes for lithium ions in the monoclinic phase of LR-Mg(st) to a greater extent than in the trigonal phase of this sample. If magnesium exists predominantly in the monoclinic phase, which transforms after activation into the manganese-containing redox species, the negative effect of the Mg doping on the kinetics of LR-Mg(st) sample (oxidation in the range of 3.0–3.3 V) is understandable. The presence of Mg^2+^ ions (additional charge) in the Li layers may hinder the re-insertion of Li^+^ ions during discharge, which results in decreasing the lithium-ion diffusion coefficient and increasing the polarization resistance, in the range of about 3 V. On the other hand, the substitution of Mg for TM in LR-Mg oxide slows the structural changes in the initial cycles and stabilizes these Li- and Mn-rich layered cathode materials.

## 4. Conclusions

Li-rich layered oxide Li_1.2_Mn_0.54_Ni_0.13_Co_0.13_O_2_ was doped with magnesium at different positions (TM and Li) using different procedures for Mg introduction. The results of XRD with Rietveld refinement, scanning and transmission electron microscopy with local EDX analysis, selected-area electron diffractions, X-ray photoelectron spectroscopy, charge–discharge tests, and galvanostatic intermittent titration showed that introduction of Mg in the TM layers is preferable to the presence of Mg at Li positions in terms of electrochemical performance. The sample doped with Mg at the TM positions (LR-Mg) shows better cyclability and an even higher discharge capacity than the reference (undoped) sample LR, at a higher current density. The poor electrochemical properties of the samples doped with Mg at Li positions (LR-Mg(st)) were due to kinetic hindrance of oxidation of manganese-containing species formed after activation of Li_2_MnO_3_ component of the composite oxide. LR-Mg(st) demonstrates the lowest lithium-ion diffusion coefficient and the greatest polarization resistance compared to LR and LR-Mg. Thus, doping with Mg has a site-dependent effect on the electrochemical behavior of Li- and Mn-rich layered cathode materials.

## Figures and Tables

**Table 1:** Refinement unit cell parameters of LR, LR-Mg, and LR-Mg(st) oxides (space group $R\bar{3}m$).

Sample	Monoclinic Phase Portion, %	*a*, Å	*c*, Å	V, Å^3^	*c*/*a*	*R*wp (%)	Goodness of Fit, S
LR	33(3)	2.84678(6)	14.2180(5)	99.788(5)	4.9944	2.81	4.12
LR-Mg	32(1)	2.84911(4)	14.2266(5)	100.011(4)	4.9933	0.91	2.87
LR-Mg(st)	31(4)	2.85207(7)	14.2379(4)	100.299(5)	4.9921	2.20	4.23

**Table 2:** Binding energies/FWHM of XP lines in the spectra of LR, LR-Mg, and LR-Mg(st) oxides.

Sample	C1s, Peak 1 ± 0.1 eV	Mn2p3 ± 0.3 eV	Co2p3 ± 0.2 eV	Ni2p3 ± 0.2 eV	Mg1s ± 0.2 eV	O1s Peak 1 ± 0.2 эB	O1s Peak 2 ± 0.2 эB
LR	284.9	642.0	779.9/1.3	854.4/1.6	-	529.3	531.0
LR-Mg(st)	284.9	642.0	779.9/1.3	854.5/1.7	1302.9/1.2	529.2	531.3
LR-Mg	284.9	642.0	779.9/1.3	854.8/1.9	1302.9/1.5	529.1	531.3

## Supplementary Materials

The following are available online at https://www.mdpi.com/article/10.3390/nano12010156/s1, Table S1: ICP-MS analysis results, Figure S1: Particle size distributions for LR, LR-Mg, and LR-Mg(st) oxides: (**a**) differential distribution; (**b**) integral distribution, Figure S2: Element distribution EDX maps of LR-Mg and LR-Mg(st) oxides (see designations on the figures).
