# Supplementary material for: Effects of Mg Doping at Different Positions in Li-Rich Mn-Based Cathode Material on Electrochemical Performance
Source: Nanomaterials (Basel). 2022 Jan 3;12(1):156. doi: 10.3390/nano12010156 (PMC8746697; doi:10.3390/nano12010156)
Supplement: Supplementary file 1 [file nanomaterials-12-00156-s001.zip › nanomaterials-1517188-supplementary.pdf]

nanomaterials

MDPI

Supplementary Materials

Effects of Mg Doping at Different Positions in Li-Rich Mn-Based Cathode Material on Electrochemical Performance

Elena Makhonina [1,*], Lidia Pechen [1], Anna Medvedeva [1], Yury Politov [1], Aleksander Rumyantsev [2], Yury Koshtyal [2], Vyacheslav Volkov [1], Alexander Goloveshkin [3] and Igor Eremenko [1,*]

1 Kurnakov Institute of General and Inorganic Chemistry, Russian Academy of Sciences, 31 Leninsky pr., 119991 Moscow, Russia; lidia.s.maslennikova@gmail.com (L.P.); anna.ev.medvedeva@gmail.com (A.M.); polserv@mail.ru (Y.P.); svolkov77@gmail.com (V.V.)
2 Ioffe Institute, Russian Academy of Sciences, 26 Politekhnicheskaya ul., 194021 St. Petersburg, Russia; rumyantsev.amr@gmail.com (A.R.); yury.koshtyal@gmail.com (Y.K.)
3 Nesmeyanov Institute of Organoelement Compounds, Russian Academy of Sciences, 28 Vavilova ul., 119334 Moscow, Russia; golov-1@mail.ru (A.G.)
* Correspondence: evma@igic.ras.ru (E.M.); ilerem@igic.ras.ru (I.E.); Tel.: +7-903-143-17-38 (E.M.)

Table S1. ICP-MS analysis results.

Sample	Determined composition	Targeted composition
LR	$Li_{1.219}Mn_{0.523}Ni_{0.129}Co_{0.129}O_2$	$Li_{1.20}Mn_{0.534}Ni_{0.133}Co_{0.133}O_2$
LR-Mg	$Li_{1.197}Mn_{0.537}Ni_{0.128}Co_{0.111}Mg_{0.027}O_2$	$Li_{1.20}Mn_{0.534}Ni_{0.133}Co_{0.113}Mg_{0.020}O_2$
LR-Mg(st)	$Li_{1.19}Mg_{0.02}Mn_{0.531}Ni_{0.130}Co_{0.129}O_2$	$Li_{1.18}Mg_{0.02}Mn_{0.534}Ni_{0.133}Co_{0.133}O_2$

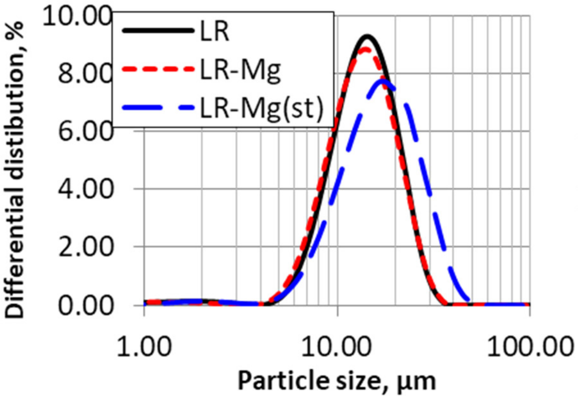

(**a**)

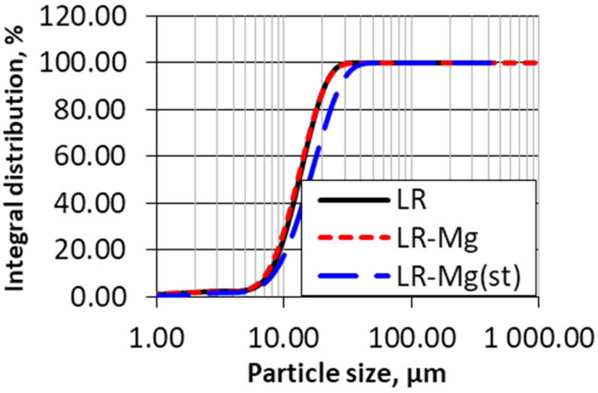

(**b**)

Figure S1. Particle size distributions for LR, LR-Mg, and LR-Mg(st) oxides: (**a**) Differential distribution; (**b**) Integral distribution.

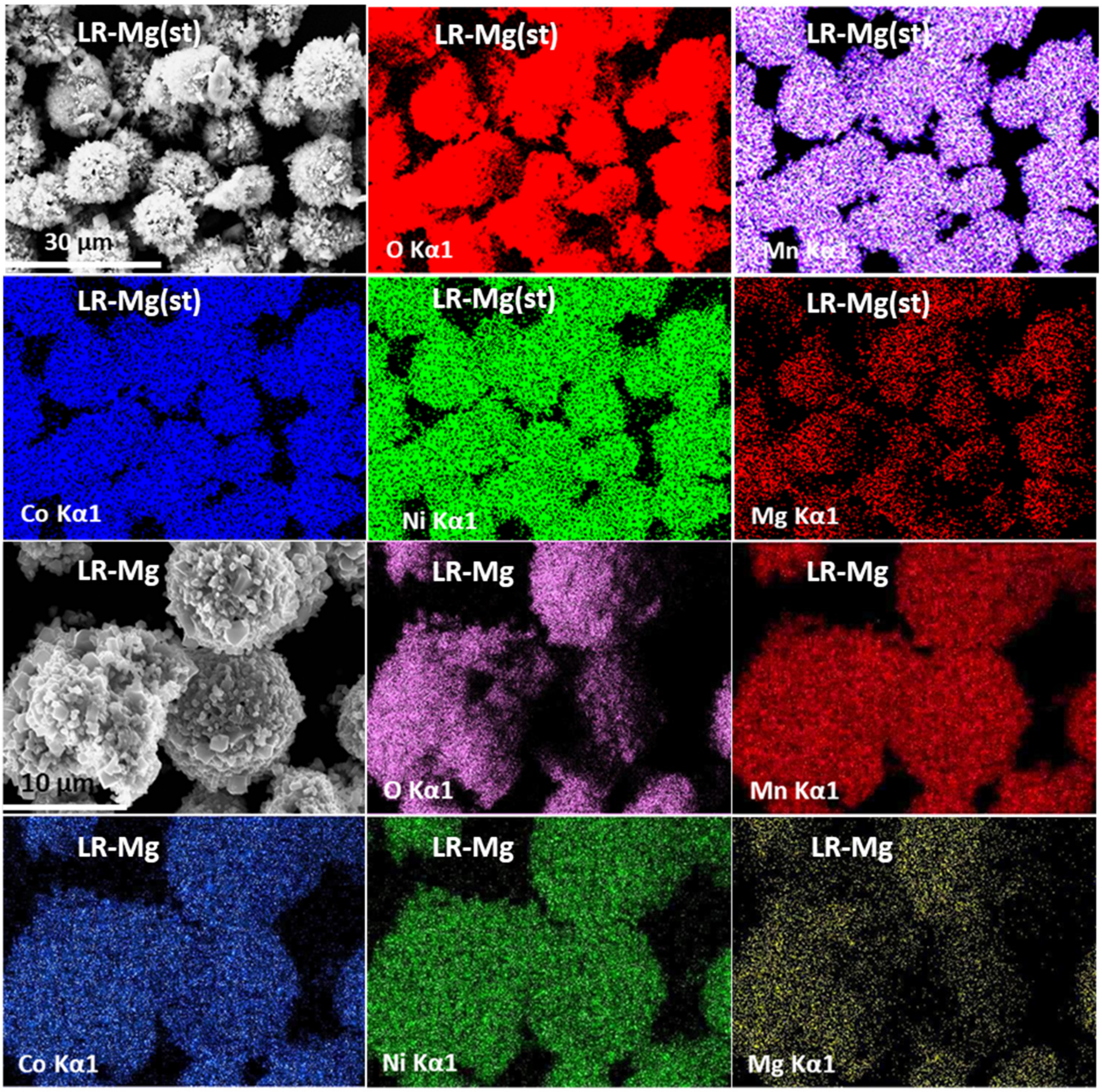

Figure S2. Element distribution EDS maps of LR-Mg and LR-Mg(st) oxides (see designations on the figures).
